# Early Life Stress Alters Expression of Glucocorticoid Stress Response Genes and Trophic Factor Transcripts in the Rodent Basal Ganglia

**DOI:** 10.3390/ijms23105333

**Published:** 2022-05-10

**Authors:** Cynthia Haidee Tran, Cynthia Shannon Weickert, Thomas Wesley Weickert, Duncan Sinclair

**Affiliations:** 1 School of Psychiatry, Faculty of Medicine, University of New South Wales, Sydney, NSW 2052, Australia; c.lee@neura.edu.au (C.H.T.); c.weickert@neura.edu.au (C.S.W.); t.weickert@neura.edu.au (T.W.W.); 2 Schizophrenia Research Laboratory, Neuroscience Research Australia, Randwick, NSW 2031, Australia; 3 Department of Neuroscience and Physiology, State University of New York, Upstate Medical University, Syracuse, NY 13210, USA; 4 Wicking Dementia Research and Education Centre, School of Medicine, University of Tasmania, Hobart, TAS 7001, Australia

**Keywords:** early life stress, brain-derived neurotrophic factor, BDNF, glucocorticoid receptor, FKBP5, dopamine

## Abstract

Early life stress shapes the developing brain and increases risk for psychotic disorders. Yet, it is not fully understood how early life stress impacts brain regions in dopaminergic pathways whose dysfunction can contribute to psychosis. Therefore, we investigated gene expression following early life stress in adult brain regions containing dopamine neuron cell bodies (substantia nigra, ventral tegmental area (VTA)) and terminals (dorsal/ventral striatum). Sprague–Dawley rats (14F, 10M) were separated from their mothers from postnatal days (PND) 2–14 for 3 h/day to induce stress, while control rats (12F, 10M) were separated for 15 min/day over the same period. In adulthood (PND98), brain regions were dissected, RNA was isolated and five glucocorticoid signalling-related and six brain-derived neurotrophic factor (*Bdnf*) mRNAs were assayed by qPCR in four brain regions. In the VTA, levels of glucocorticoid signalling-related transcripts differed in maternally separated rodents compared to controls, with the *Fkbp5* transcript significantly lower and *Ptges3* transcript significantly higher in stressed offspring. In the VTA and substantia nigra, maternally separated rodents had significantly higher *Bdnf* IIA and III mRNA levels than controls. By contrast, in the ventral striatum, maternally separated rodents had significantly lower expression of *Bdnf I*, *IIA*, *IIC*, *IV* and *VI* transcripts. Sex differences in *Nr3c1*, *Bag1* and *Fkbp5* expression in the VTA and substantia nigra were also detected. Our results suggest that early life stress has long-lasting impacts on brain regions involved in dopamine neurotransmission, changing the trophic environment and potentially altering responsiveness to subsequent stressful events in a sex-specific pattern.

## 1. Introduction

Environmental stress, defined as an aversive external event in which a compensatory response is triggered to maintain an individual’s homeostasis [1], has profound and long-lasting effects on the mammalian brain [2]. For example, early life stress shapes the developing brain by altering expression of genes and proteins in the cortex and hippocampus [3,4,5,6] and results in enduring dysregulation of stress hormone secretion via changes in the hypothalamic–pituitary–adrenal (HPA) axis [7,8,9]. Importantly, early life stress increases risk for anxiety, post-traumatic stress disorder and psychotic disorders such as schizophrenia [10,11,12,13,14].

**Figure 1:**
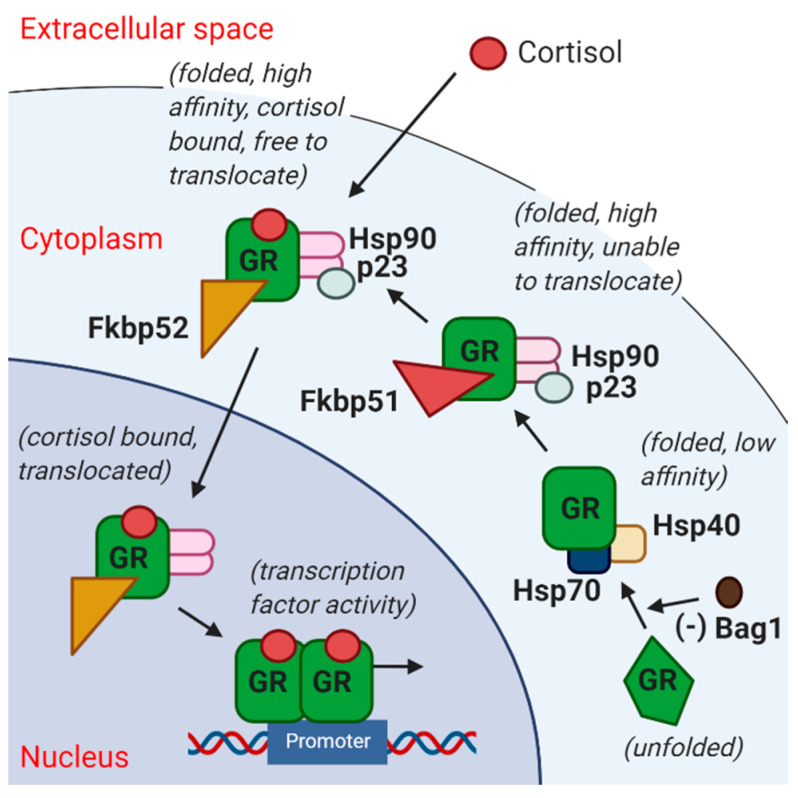
Simplified diagram of the glucocorticoid receptor (GR) stress signalling pathway. GR (unfolded) is unable to bind cortisol when bound to Bag1, and release of Bag1 allows GR to bind cortisol with low affinity, whereas Fkbp51 (encoded by *Fkbp5*) and p23 (encoded by *Ptges3*) are involved in increasing GR affinity to cortisol by stabilizing the GR heterocomplex into a high affinity state. Furthermore, Fkbp52 (encoded by *Fkbp4*) dislocates Fkbp51 and facilitates nuclear translocation of the cortisol-bound GR heterocomplex into the nucleus to activate or repress target genes. Thus, higher Fkbp51 would promote GR retention in the cytoplasm, rendering target genes less responsive to stress [16,17,18,19]. GR = glucocorticoid receptor; Hsp = heat shock protein.

Responses to early life stress are mediated by glucocorticoid stress hormones, which regulate cellular adaptation to environmental stimuli and play a key role in health and disease [15]. Molecules involved in intracellular glucocorticoid signalling (Figure 1) include the glucocorticoid receptor (GR; encoded by the *Nr3c1* gene), Bag1, p23 (encoded by the *Ptges3* gene), Fkbp51 (encoded by the *Fkbp5* gene) and Fkbp52 (encoded by the *Fkbp4* gene) [16,17,18,19]. Brain expression levels of GR (*Nr3c1*) and *Fkbp5* are regulated by stress hormones, as part of the negative feedback control of HPA axis activity [20,21,22,23]. In the postmortem brain and peripheral blood of people with schizophrenia, the glucocorticoid stress-signalling pathway is dysregulated and this dysregulation is linked to positive, negative and general symptom severity in schizophrenia and to mood in schizoaffective disorder [24,25,26]. Here, we hypothesised that early life stress would result in enduring gene expression changes, which would be predicted to diminish cellular glucocorticoid responsiveness, akin to changes seen in the brains of individuals with psychotic disorders—specifically a decrease in *GR* mRNA and an increase in *FKBP5* mRNA [24,25].

Brain-derived neurotrophic factor (BDNF) is an important trophic factor whose abundance is modulated by glucocorticoids [27,28]. It is found throughout the brain, including within the midbrain and (at low levels) the striatum [29,30]. Cross-talk between BDNF and glucocorticoid signalling is thought to be vital for programming later-life stress responsiveness [31]. Dopamine neurons are particularly responsive to BDNF, and BDNF prolongs the survival of cultured dopamine neurons [32,33,34,35]. BDNF modulates synaptic function, synaptic plasticity, learning and memory throughout development and maturation [34,35,36,37]. Prolonged maternal separation in rodents has been reported to decrease hippocampal *Bdnf* mRNA and protein during or immediately after the period of separation [38,39] with changes persisting into adulthood (reviewed in [31]). Maternal separation is also reported to decrease striatal BDNF protein in adulthood [40]. Some studies have reported increased BDNF in adult animals exposed to early life stress, and this inconsistency may be explicable, in part, by the expression of numerous BDNF transcripts in the brain. The rat *Bdnf* gene consists of nine exons (eight 5′ untranslated exons and one common 3′ protein coding exon [30]) with different *Bdnf* transcripts formed through the use of different promoters [41,42]. Expression of distinct *Bdnf* transcripts varies between brain regions [30,41] and in response to stress (reviewed in the hippocampus in [31]). BDNF signalling in the mesolimbic dopamine pathway is believed to play a major role in stress-related behaviours including responses to social defeat stress [43,44]. Reductions in distinct BDNF transcripts in the DLPFC (*BDNF II*), parietal cortex (*BDNF II* and *IV*) and hippocampus (*BDNF VI*) occur in schizophrenia [45]. Thus, we hypothesised that early life stress downregulates rat *Bdnf* gene expression in adulthood.

Not only are anxiety, post-traumatic stress disorder and schizophrenia stress-related disorders, but they all involve changes to dopaminergic signalling pathways [46,47,48,49,50,51]. As a result, an understanding of how early life stress impacts the molecular landscape of stress signal transducers and trophic factors in regions where dopamine cell bodies reside and where dopamine neurotransmission is enriched is warranted. The midbrain and striatum are key nodes of dopamine neurotransmission and neuroregulation. Two major subcortical dopaminergic pathways in the rodent brain include the nigrostriatal pathway (substantia nigra to dorsal striatum) and the mesolimbic pathway (ventral tegmental area (VTA) to ventral striatum) [52,53]. Brain dopamine is most concentrated at the cell body regions in the substantia nigra and VTA [53,54] and at terminal regions in the striatum. Aversive stimuli or stressors have been shown to heighten dopaminergic activity in the adult human VTA, dorsal striatum and ventral striatum (reviewed in [55,56]). Since dopaminergic brain regions are activated by stressful stimuli, it is plausible that the molecular changes in stress pathways or trophic support in the areas of dopamine cell bodies and terminals are permanently changed by early life stress, which may have important implications for the later-life emergence of neuropsychiatric disorders.

Although stress in adulthood increases dopamine levels in the brain (reviewed in [55,56]), little is known about how early life stress may impact the molecular environment of dopamine neurons. The present study evaluated the long-lasting effects of early life stress on gene expression in dopaminergic regions in rats, with a focus on transcripts encoding proteins that are stress-sensitive and that also have important trophic functions for neuronal plasticity and viability.

We hypothesised that early life stress alters expression of stress-signalling factors (*Nr3c1*, *Fkbp4*, *Fkbp5*, *Ptges3*, *Bag1*) and splice variants of *Bdnf* in such a way as to decrease the sensitivity of dopaminergic regions to glucocorticoid (stress) hormones and diminish trophic support for dopaminergic neurons, at the transcriptional level. Since stress and sex hormones interact to influence dopaminergic brain regions (reviewed in [57]) and females are less vulnerable to dopamine-related psychopathology than males [58,59], we also hypothesised that transcriptional changes due to early life stress would occur in a sex-dependent way with females poised to be more resilient to stress than males.

## 2. Results

### 2.1. Expression of Stress-Related Factors in the Substantia Nigra, Ventral Tegmental Area and Dorsal and Ventral Striatum

#### 2.1.1. Effects of Early Life Stress on Expression of Stress-Related Transcripts, Seen in Both Females and Males

**Figure 2:**
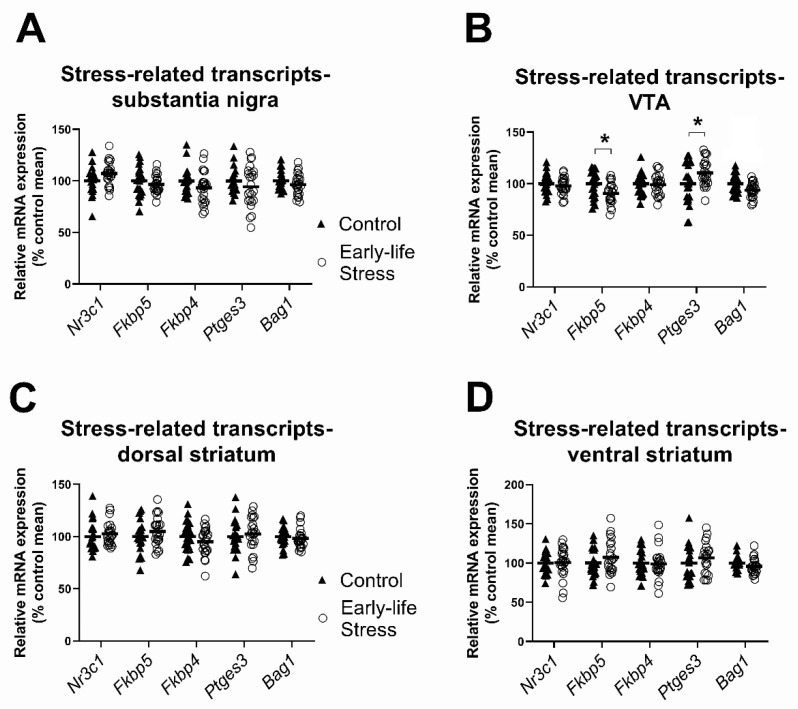
Effects of early life stress on stress-related transcript levels. (**A**,**C**,**D**) There were no main effects of early life stress in the substantia nigra, dorsal striatum or ventral striatum; (**B**) maternally separated rats of both sexes had lower *Fkbp5* and higher *Ptges3* mRNA in the VTA than control rats. Data are expressed as a percentage of the control group mean. Each data point represents a single animal. Filled triangles and hollow circles represent control and early life stress animals, respectively. Horizontal lines depict group means. * *p* < 0.05.

In the substantia nigra, there were no significant main effects of early life stress on expression levels for any of the five stress-related transcripts of interest (all F ≤ 3.78, all *p* ≥ 0.06; see Figure 2A, Table 1).

In the VTA there was a significant main effect of early life stress on levels of two stress-related transcripts in the VTA, *Fkbp5* and *Ptges3* (see Figure 2B, Table 1). Opposite to our prediction, mRNA expression levels for *Fkbp5* was significantly lower (by 10%) in the maternally separated group compared to the control group. By contrast, levels of *Ptges3* mRNA were 10% higher in the VTA of the maternally separated group compared to controls.

In the dorsal and ventral striatum, there were no significant main effects of early life stress (all F ≤ 2.60, all *p* ≥ 0.11; see Table 1) for any of the stress-related transcripts measured.

#### 2.1.2. Sex Differences in the Effects of Early Life Stress on Expression of Stress-Related Transcripts

**Figure 3:**
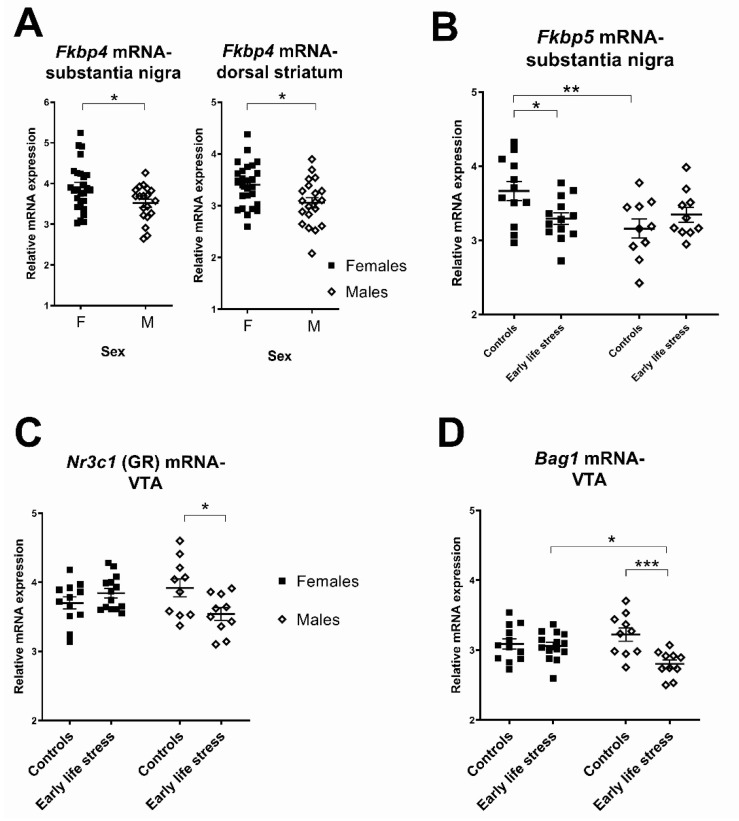
Differences between females and males in stress-related transcript levels and effects of early life stress. (**A**) Females had significantly higher *Fkbp4* mRNA levels in the substantia nigra and dorsal striatum than males; (**B**) female control rats had higher *Fkbp5* mRNA levels in the substantia nigra than male control rats. They also had higher *Fkbp5* mRNA levels than maternally separated female rats; (**C**) maternally separated male rats had lower *Nr3c1* (GR) mRNA levels in the VTA than male control rats; (**D**) maternally separated male rats had lower *Bag1* mRNA levels in the VTA than male control rats. Each data point represents a single animal. Filled squares and hollow diamonds represent female and male animals, respectively. Horizontal lines depict group means. * *p* < 0.05, ** *p* < 0.005, *** *p* < 0.0005.

In the substantia nigra, there were significant main effects of sex on two stress-related transcripts: *Fkbp5* and *Fkbp4* (F’s ≥ 4.17, *p*’s ≤ 0.05). Compared to male rats, female rats had higher *Fkbp5* transcript levels and higher *Fkbp4* levels (Figure 3A,B, 7–11%) in the substantia nigra. There were no significant main effects of sex on *Nr3c1*, *Ptges3* or *Bag1* mRNAs (all F ≤ 2.33, all *p* ≥ 0.14). Additionally, for the *Fkbp5* transcript there was a significant interaction between sex and early life stress in the substantia nigra (Table 1). Female control rats had higher *Fkbp5* mRNA levels than male control rats (*p* < 0.005, Figure 3B) and maternally separated female rats (*p* < 0.05). For all other genes of interest, there were no significant interactions between sex and early life stress (all F ≤ 1.19, all *p* ≥ 0.28) in the substantia nigra.

In the VTA there were significant interactions between sex and early life stress for two stress-related transcripts, *Nr3c1* and *Bag1* (Table 1). Compared to control male rats, maternally separated male rats had lower *Nr3c1* mRNA as predicted (10% (*p* = 0.009); Figure 3C), and also had lower *Bag1* mRNA (13% (*p* < 0.001); Figure 3D). For these transcripts, effects were not seen in females. For all other transcripts of interest in the VTA, there were no significant interactions between sex and early life stress (all F ≤ 1.13, all *p* ≥ 0.29).

In the dorsal striatum, there was a significant main effect of sex on *Fkbp4* mRNA expression, such that female rats had 12% higher *Fkbp4* mRNA expression than male rats (see Figure 3A). There were no other significant main effects of sex on any other transcripts of interest in the dorsal striatum (all F ≤ 0.36, all *p* ≥ 0.55). Similarly, there were no significant main effects of sex on any transcripts of interest in the ventral striatum (all F ≤ 1.77, all *p* ≥ 0.19; see Table 1). There were no significant interactions between sex and early life stress for any stress-related transcripts of interest in the dorsal striatum (all F ≤ 2.07, all *p* ≥ 0.16) or ventral striatum (all F ≤ 2.78, all *p* ≥ 0.10; see Table 1).

### 2.2. Expression of Bdnf Transcripts in the Substantia Nigra, Ventral Tegmental Area and Dorsal and Ventral Striatum

#### 2.2.1. Effects of Early Life Stress on Expression of *Bdnf* Transcripts, Seen in Both Females and Males

**Figure 4:**
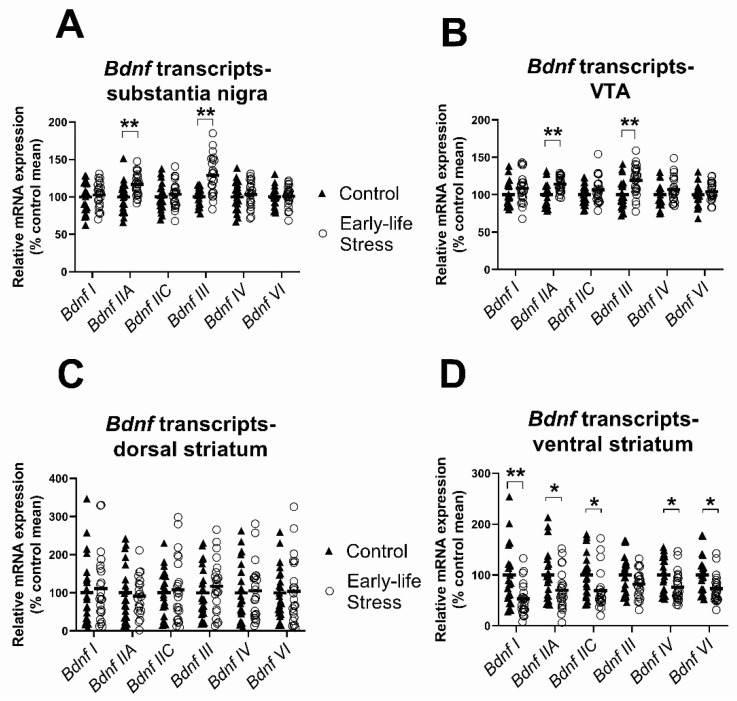
Effects of early life stress on BDNF transcript levels. (**A**,**B**) Maternally separated rats had higher levels of *Bdnf IIA* and *III* mRNAs in the substantia nigra and VTA than control rats; (**C**) there were no main effects of early life stress on *Bdnf* transcripts in the dorsal striatum; (**D**) maternally separated rats had lower levels of *Bdnf I, IIA, IIC, IV* and *VI* mRNAs in the ventral striatum than control rats. Data are expressed as a percentage of the control group mean. Each data point represents a single animal. Filled triangles and hollow circles represent control and early life stress animals, respectively. Horizontal lines depict group means. * *p* < 0.05, ** *p* < 0.005.

There was a significant main effect on early life stress on *Bdnf IIA* and *Bdnf III* mRNA levels (see Figure 4, Table 2). Contrary to our expectation, mRNA expression of both *Bdnf* transcripts was increased in the substantia nigra of maternally separated rats of both sexes compared to controls (*Bdnf IIA*: 17% and *Bdnf III*: 29% increase; see Figure 4A). For the remaining 4 *Bdnf* transcripts measured, there were no significant main effects of early life stress in the substantia nigra (all F ≤ 0.51, all *p* ≥ 0.48).

Identical to the stress-related pattern of changes detected in the adjacent substantia nigra, we detected a significant main effect of early life stress on two *Bdnf* transcripts, *Bdnf IIA* and *Bdnf III* in the VTA (see Table 2). These two *Bdnf* transcripts were higher (*Bdnf IIA*: 14% and *Bdnf III*: 20%) in the VTA of the maternally separated group compared to controls (see Figure 4B). In all other *Bdnf* transcripts, there were no significant main effects of early life stress (all F ≤ 2.02, all *p* ≥ 0.16).

In the dorsal striatum, there were no significant main effects of early life stress (all F ≤ 0.45, all *p* ≥ 0.51, Figure 4C) for any *Bdnf* transcript.

In the ventral striatum, there were robust and widespread significant main effects of early life stress on five *Bdnf* transcripts: *Bdnf I, Bdnf IIA, Bdnf IIC, Bdnf IV* and *Bdnf VI* (all F ≥ 5.48, all *p* ≤ 0.02; see Table 2). Interestingly, in contrast to the midbrain, but consistent with our hypothesis, maternally separated rodents had lower, rather than higher, mRNA levels than control rodents for the five *Bdnf* transcripts in the ventral striatum (*Bdnf I*: 46%, *Bdnf IIA*: 15%, *Bdnf IIC*: 31%, *Bdnf IV*: 26% and *Bdnf VI*: 26%, see Figure 4D). For *Bdnf III*, there were no significant main effects of early life stress in the ventral striatum.

#### 2.2.2. Sex Differences in the Effects of Early Life Stress on Expression of Bdnf Transcripts

**Figure 5:**
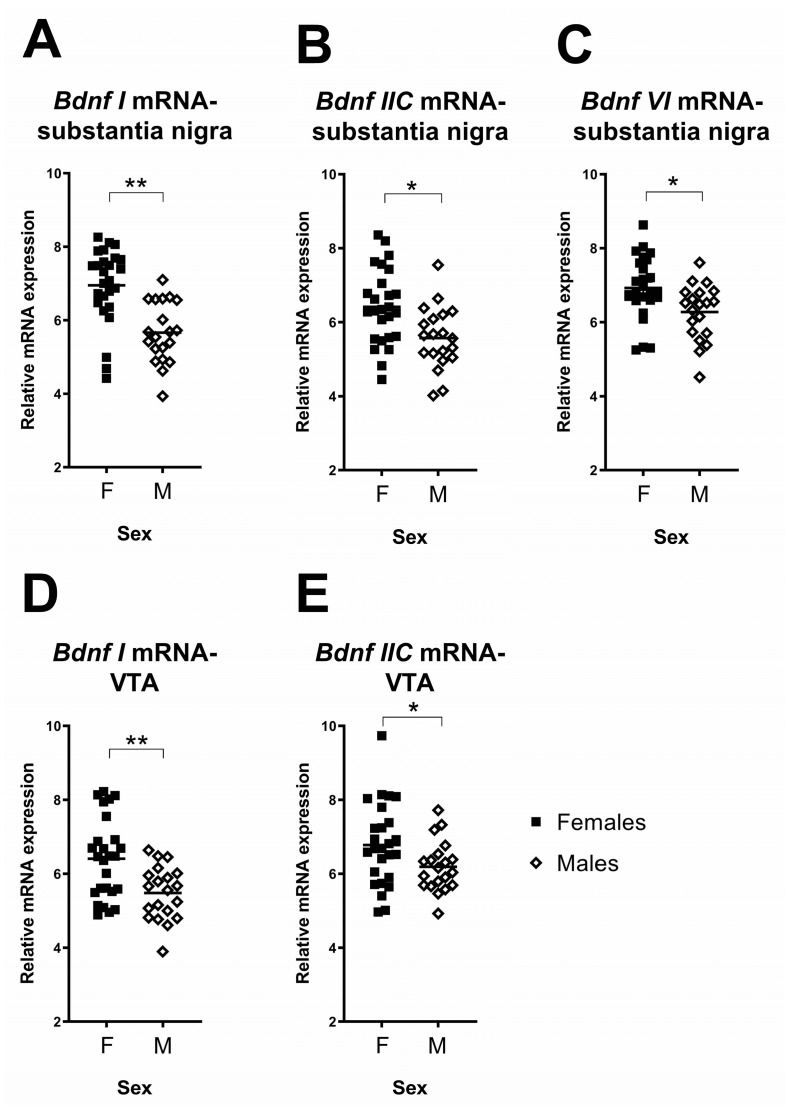
Differences between females and males in *Bdnf* transcript levels. (**A**–**C**) Females had significantly higher levels of *Bdnf I, IIC* and *VI* in the substantia nigra than males; (**D**,**E**) females had significantly higher levels of *Bdnf I* and *IIC* in the VTA than males. Each data point represents a single animal. Filled squares and hollow diamonds represent female and male animals, respectively. Horizontal lines depict group means. * *p* < 0.05, ** *p* < 0.005.

In the substantia nigra there was a significant main effect of sex on three *Bdnf* transcripts: *Bdnf I, Bdnf IIC* and *Bdnf VI* (all F ≥ 7.12, all *p* ≤ 0.01; see Table 2). In all cases, female rats had higher (*Bdnf I*: 23%, *Bdnf IIC*: 15% and *Bdnf VI*: 10%) mRNA expression than male rats (see Figure 5A–C). There were no significant main effects of sex in *Bdnf IIA*, *Bdnf III* or *Bdnf IV* (all F ≤ 3.11, all *p* ≥ 0.09). For all Bdnf transcripts in the substantia nigra, there were no significant interactions between sex and early life stress (all F ≤ 2.28, all *p* ≥ 0.14).

In the VTA, similar to the substantia nigra, there was a significant, but subtler, main effect of sex on two changed *Bdnf* transcripts, *Bdnf I* and *Bdnf IIC* mRNAs (see Table 2), such that female rats had higher *Bdnf I* (9%) and *Bdnf IIC* (9%) mRNA in the VTA than male rats (see Figure 5D,E). There were no further significant main effects of sex in the other *Bdnf* transcripts (all F ≤ 1.79, all *p* ≥ 0.19). For all *Bdnf* transcripts in the VTA, there were no significant interactions between sex and early life stress (all F ≤ 3.17, all *p* ≥ 0.08).

In the dorsal striatum there were no main effects of sex or interactions between sex and early life stress for *Bdnf* transcripts (all F ≤ 2.58, all *p* ≥ 0.12; see Table 2).

Similarly, in the ventral striatum there were no significant main effects of sex (all F ≤ 2.09, all *p* ≥ 0.16), or early life stress–sex interactions (all F ≤ 1.22, all *p* ≥ 0.28) for any *Bdnf* transcripts.

## 3. Discussion

In this study, we found region-specific, sex-specific and transcript-specific differences in how prolonged early life stress (maternal separation) impacts gene expression in mesolimbic and nigrostriatal regions of the rat brain. Sensitivity to early life stress varied between brain regions—changes in the levels of stress-signalling transcripts were found in the midbrain (substantia nigra and VTA) but not in the striatum. Changes to the levels of *Bdnf* transcripts were found in the substantia nigra, VTA and the ventral striatum, but not in the dorsal striatum. Thus, the mesolimbic pathway may be more vulnerable to long-term stress-induced changes overall as compared to the nigrostriatal pathway.

Previous research in mice and rats using the early life stress paradigm employed here (3 h prolonged separation vs. 15 min brief separation controls) has shown that prolonged early life stress leads to increased depression-relevant behaviours, when compared to brief separation controls [60,61]. In this paradigm, greater depression-relevant behaviour has been reported in male rats exposed to early life stress compared to females [62]. Increased anxiety-related behaviours have also been reported in prolonged early life stress rats compared to brief separation controls [61,62]. These findings are consistent overall with those of studies using animal facility reared controls (reviewed in [63]).

Region-specific sex differences were seen in stress-naïve control animals in our study, which may contribute to differences in stress sensitivity in adulthood. Expression of *Fkbp4*, which constrains GR nuclear translocation when bound in the GR–chaperone complex [64], was greater in the dorsal striatum in females than in males. This may make the dorsal striatum less stress hormone responsive in females. In the substantia nigra, sex differences in stress-naïve control animals may counter-balance each other—control females had greater expression of both *Fkbp4* and *Fkbp5*, two GR chaperones with opposing effects on nuclear translocation [64].

We hypothesised that males would experience greater impacts of early life stress than females, in line with previous studies reporting that female Sprague–Dawley rats were more resilient to behavioural effects of early life stress than males [62,65]. We observed sex differences due to early life stress that were only partially consistent with this hypothesis—fewer transcripts were altered by early life stress in the VTA in females than males, but in females (but not males) impacts of early life stress were seen in the substantia nigra. In females, our findings suggest that early life stress may alter the midbrain to increase stress hormone responsiveness later in life. Early life stress decreased *Fkbp5* and *Ptges3* mRNA expression in the VTA in females, and also decreased *Fkbp5* mRNA expression in the substantia nigra. These changes could make neurons more responsive to cortisol by promoting the binding of cortisol to the high-affinity GR heterocomplex and/or promoting translocation of GR into the nucleus [64,66]. In males, a more diverse pattern of stress-induced gene expression changes was seen specifically in the VTA. *Fkbp5*, *Ptges3*, *Nr3c1* (GR) and *Bag1* mRNAs all decreased following early life stress. Decreased GR mRNA in the VTA of males may diminish cortisol responsiveness. However, since Bag1 protein inhibits GR activation, decreased *Bag1* expression would be predicted to increase cortisol sensitivity. Decreased *Fkbp5* and *Ptges3* would likewise be expected to increase cortisol sensitivity. While the net effect of these combined molecular changes on stress responsiveness is not clear, males appear to have a more complex molecular response to early life stress in the VTA than females. Overall, our findings suggest that the molecular regulation of the adult stress response may be fundamentally altered after early life stress in males. It is plausible that decreased *Fkbp5* may represent an adaptive mechanism, since decreased *Fkbp5* expression has been observed in resilient mice in a model of PTSD [67], *Fkbp5*-knockout mice display behavioural stress resilience [68] and distinct *FKBP5* methylation patterns have been associated with post-traumatic growth in humans [69].

The trophic environment may also be impacted by developmental stress in a region-specific manner. Selected *Bdnf* transcripts were increased in maternally separated rodents in the midbrain, while almost all *Bdnf* transcripts were decreased in the ventral striatum. *BDNF* mRNA has been found within human dopamine neurons [70], so it is possible that after early life stress, more BDNF, which is synthesised in the midbrain, would be available for local neurotrophic support at the level of the dopamine neuronal cell body and dendrites. This differs from target-derived neurotrophic support (i.e., BDNF produced and secreted by cells in the striatum, taken up by the axon terminal and retrogradely transported to the midbrain), which from our study, would be expected to be diminished when emanating from the ventral striatum by early life stress. Our findings are only partially consistent with our hypothesis that early life stress would decrease *Bdnf* expression, since lower *Bdnf* mRNA was found in only one of four regions examined. While this is, to our knowledge, the first study of early life stress on *Bdnf* gene expression in the midbrain, another study has reported decreased mature BDNF protein in the VTA following early life stress [71]. Since *BDNF* expression is highly regulated by neuronal activity [72], and early life stress can cause hyperexcitability of VTA dopamine neurons [73], it is plausible that regional changes in *Bdnf* mRNA expression may relate to long-lasting stress-induced changes in the excitability of those regions. In sum, while it is possible that the total BDNF available to the dopamine neurons is unaltered after early life stress in normal rodents, the anatomical source of BDNF may change following stress exposure and this putative change could have consequences on the strength and plasticity of connections within the mesolimbic circuitry following early life stress.

Not only did we detect region-specific changes, but we also detected transcript-specific changes following early life stress on *Bdnf* expression. In our study, only *Bdnf IIA* and *III* were increased in the midbrain following early life stress. Increased *Bdnf III* and *Bdnf VI* have been reported in the hippocampus of separated pups immediately following material separation or months later in adulthood [39,74] with changes in *Bdnf VI* found after early life or adult stress [74,75]. Interestingly, transcript-specific impacts of adult stress may be modifiable by early life stress exposure [74]. Not all effects of early life stress in our study were transcript-specific, since all *Bdnf* transcripts were decreased following early life stress in the ventral striatum and these *Bdnf* reductions were the most robust transcriptional change detected in our study. Since many of the dopamine neurons projecting to the VTA co-release glutamate and glutamate is a powerful inducer of *Bdnf* mRNA [76], the reduction in *Bdnf* expression could reflect less activity of mesoaccumbens projections in adulthood after early life stress.

We observed sex differences in *Bdnf I* and *Bdnf IIC* such that female rats had higher *Bdnf* mRNA expression in the midbrain than male rats irrespective of stress exposure. While we did not observe sex–stress interactions, previous studies reported that males experience greater decreases in hippocampal BDNF [77] and hypothalamic BDNF [78] following early life stress as compared to females. Another study reported increases in hippocampal *Bdnf I* and *IV* in females but not males following prenatal stress [79]. This diversity of findings is not unexpected, since the effects of stress on BDNF levels differ depending on the strain of rodents, the sex of the animal, the type of stress and brain region examined [31,74]. Taken together it appears that most BDNF promoters can be stress-responsive in the brain depending on context. The higher baseline BDNF expression in the midbrain of females in our study may suggest that dopamine neurons, which depend on BDNF for trophic support, may be more resilient to insults in adult females compared to males, regardless of early life stress exposure, or that activity-dependent expression of BDNF is increased in females compared to males.

There are some limitations to this study. For ethical reasons, only four litters were used, split between two experimental conditions, which minimised the number of animals bred and stressed unnecessarily while still ensuring an appropriate sample size of offspring. As a result, it was difficult to control for litter effects that arise due to changes in the mother’s behaviour towards the pups following maternal separation [80]. Other studies have employed one 24 h maternal separation period, which may result in greater behavioural impairment, but this model can be variable and strain-dependent [81,82,83,84]. The 3 h per day maternal separation model of early life stress in rodents chosen in this study has been shown to induce molecular, hormonal and behavioural changes akin to those induced by early life stress in humans [60,85,86]. Lastly, we did not quantify proteins in this study, so the functional significance of our observations would depend on whether gene expression changes were reflected in expected changes at the protein level, but since many of the key factors assayed in this study are regulated at the transcriptional level, we think this is likely.

In conclusion, we found that early life stress induced long-lasting changes in glucocorticoid stress signalling and *Bdnf* transcripts in adult midbrain areas. These changes differed according to brain region, transcript and sex, highlighting complexities in the long-term brain response to early life stress. Regions of origin and termination of mesolimbic dopamine neurons appeared more sensitive to early life stress than regions of origin and termination of nigrostriatal dopamine neurons. Stress-related and *Bdnf* transcripts demonstrated sex-specific expression patterns that are suggestive of neuroprotection in females. Future work may shed light on whether early life stress-induced changes in the midbrain result in altered dopaminergic response to stress later in life, and whether these may increase risk for dopamine dysregulation and behavioural changes relevant to psychotic mental illness.

## 4. Materials and Methods

### 4.1. Experimental Design

Four timed-pregnant female Sprague–Dawley rats (Animal Resource Centre, Canning Vale, WA, Australia) were supplied to Neuroscience Research Australia before gestational day 17. Rats were housed in 57 cm × 35 cm × 19 cm polypropylene cages containing nesting materials on 12 h light:12 h dark cycles at 23 °C. Standard rat chow and water were available ad libitum. After birth, pups were housed one litter per cage with their respective dams.

The experimental group (2 litters composed of 24 pups (14 F, 10 M)) received extended (3 h) maternal separation while the control group (2 litters composed of 22 pups (12 F, 10 M) received only a brief (15 min) maternal separation as per a previously published protocol [61]. For the control group, brief 15 min separation was chosen to standardize handling between experimental groups and minimize variability associated with the use of animal facility reared controls. Brief 15 min separation is not considered aversive (reviewed in the context of ethanol consumption in [87]) and was used as a control group in other studies [60,61,62,88,89,90]. The use of a brief separation control is also more naturalistic than an animal facility reared control, as it mimics animal behaviour in the wild. For ethical reasons, to limit culling of unused animals, the minimum possible number of litters was used to achieve the necessary sample size. On each day of maternal separation, the dam was removed from their home cage between 13:00 and 15:00 and housed on their own in a new cage. The litter was placed in a small box containing bedding pellets and shredded paper on a heating mat at 32 °C in a separate, but adjacent room for either 15 min (control group) or 3 h (stress group). Daily maternal separation occurred from postnatal day 2 to 14 inclusive. Dams were returned to their cages after return of their pups.

On postnatal day 20, all pups were weaned and housed with same sex littermates (2–5/cage) on 12 h light:12 h dark cycles at 23 °C. Standard rat chow and water were available ad libitum. At postnatal day 98, rats were anaesthetised by intraperitoneal injection of pentobarbitone and euthanized by decapitation (guillotine). The brain was extracted from the skull and parts of the brain (i.e., substantia nigra, VTA, dorsal striatum and ventral striatum) were dissected, following the Rat Brain Atlas [91] as a guide. For the VTA and substantia nigra, the midbrain block was cut squarely at the cerebral aqueduct and then triangularly to form tissue chunks with the medial VTA and two adjacent lateral chunks containing the left and right substantia nigra. Samples were frozen on foil on dry ice and then placed individually in 1.5 mL RNase-free Axygen microtubes (Axygen, Union City, CA, USA) and stored at −80 °C until the day of RNA extraction.

### 4.2. RNA Extraction

Total RNA was extracted from 46 maternally separated rats (24 stressed (14 F, 10 M) and 22 control rats (12 F, 10 M)) in four brain regions of interest: substantia nigra (left), ventral tegmental area (medial), dorsal striatum (left) and ventral striatum (left). Rodent brain tissue (~30–100 mg) was homogenised manually in 800 μL of TRIzol reagent (Life Technologies, Carlsbad, CA, USA) using a pestle, in a 1.5 mL RNase-free Axygen microtube (Axygen, Union City, CA, USA) and incubated for 5 min at room temperature. Chloroform (160 μL) was added to the homogenized samples and vortexed until completely mixed. The sample was centrifuged at 12,000× *g* for 15 min at 4 °C. Supernatant was transferred into new 1.5 mL RNase-free tubes. Four hundred microlitres of 100% 2-propanol (Sigma-Aldrich, Saint Louis, MO, USA) was added to the supernatant and the tube was inverted 10 times and incubated at room temperature for 10 min. Samples were centrifuged at 12,000× *g* for 10 min at 4 °C. The supernatant was removed, leaving the RNA pellet on the bottom of the tube. The RNA pellet was washed with 800 μL of 70% ethanol and centrifuged at 7500× *g* for 5 min at 4 °C. The ethanol wash was discarded and the RNA pellet was left to air dry. When the pellet was clear, the RNA pellet was resuspended in 30 μL of RNase-free water for the ventral striatum, and 50 μL of RNase-free water for the dorsal striatum, substantia nigra and VTA. RNA concentration was quantified using a ND-1000 Spectrophotometer (Nanodrop Technologies, Wilmington, DE, USA). RNA integrity was checked, using a high-resolution capillary electrophoresis in a subset of samples (Agilent Bioanalyzer 2100, Agilent Technologies, Palo Alto, CA, USA). RNA Integrity Numbers were greater than or equal to 8.3 for all samples tested.

### 4.3. cDNA Synthesis

RNA (2 μg per sample) was reverse-transcribed to cDNA using SuperScript IV First-Strand cDNA synthesis reaction and random hexamers, following the manufacturer’s protocol (Life Technologies, Ref #18091050). Alongside sample reactions, a negative control (without reverse transcriptase) and a no template control (without RNA) were included.

### 4.4. Quantitative Polymerase Chain Reaction (qPCR)

Real-time PCR was performed in triplicate using Taqman Gene Expression Assays (Applied Biosystems, Foster City, CA, USA) and an ABI Prism 7900HT Fast Real-Time PCR System. The PCR mix was made using a master mix (17.5 μL), a probe of interest (1.75 μL) and DEPC-treated water (5.25 μL). The PCR mix (24.5 μL) was dispensed into each well of the 96-well plate and mixed thoroughly by pipetting up and down 10 times. Ten microlitres of the solution, containing sample and PCR mix, were transferred from the 96-well plate into duplicate wells of a clean 384-well plate. The probesets used were: *Nr3c1* (GR)-Rn00561369_m1; *Fkbp5*-Rn01768371_m1; *Fkbp4-*Rn01459299_m1; *Ptges3*-Rn01529546_m1; Bag1-Rn01439834_m1; *Bdnf I-*Rn01484924_m1; *Bdnf IIA*-Rn00560868_m1; *Bdnf IIC*-Rn01484925_m1; *Bdnf III*-Rn04230563_m1; *Bdnf IV*-Rn01484927_m1; *Bdnf VI*-Rn01484928_m1. Reactions were performed in triplicate and the mean relative quantity was calculated using the standard curve method. To generate the seven-point standard curve, pooled cDNAs from a subgroup of 16 rodents per region, including samples from the control and stressed groups, were serially diluted. A negative control (without reverse transcriptase) and a no template control (NTC) did not show a signal for any mRNA. The PCR cycling conditions were 50 °C for 2 min, 95 °C for 10 min, 45 cycles of 95 °C for 15 s and 60 °C for 1 min. Sequence Detector Software Version 2.4 (Applied Biosystems) was used to record real-time fluorescence intensity, while the threshold was located within the linear ranges of the amplification for each primer/probe target set.

### 4.5. Statistical Analyses

Stress-related and *Bdnf* mRNAs were normalized to the geomean of three housekeeper genes: *GusB* (probeset Rn00566655_m1), 18s (probeset Hs99999901_s1), *Ywhaz* (probeset Rn00755072_m1). The geometric means of the mRNAs used for normalisation did not differ significantly between early life stress conditions, and thus appeared stable (all brain regions t ≤ 2.0, df = 44, *p* ≥ 0.06).

Within each treatment (early life stress and control) group, outliers were identified using the Grubbs Test iteratively. Normal distribution of the data was investigated using QQ plots. Non-normally distributed data were log-transformed and again checked for normal distribution. Two-way ANOVAs were performed to assess for a main effect of treatment or sex on mRNA normalised expression levels of stress-related and *Bdnf* transcripts in the four brain regions of interest, or a sex–treatment interaction. Least Significant Difference (LSD) post hoc tests were used following any significant interactions. All analyses were performed using Statistical Package for the Social Sciences (SPSS, Armonk, New York, NY, USA) Edition 24. For all analyses, the alpha level set at *p* ≤ 0.05.

## Figures and Tables

**Table 1:** Two-way ANOVA showing main effects of early life stress and sex, as well as interactions between early life stress and sex, on stress signalling-related transcripts in the substantia nigra, VTA, dorsal striatum and ventral striatum of 22 control and 24 maternally separated rodents. Asterisks (*) indicate genes whose log-transformed data were used for statistical analysis. VTA = ventral tegmental area; GR = glucocorticoid receptor. Bold type indicates statistically significant effects.

Transcript	Outliers (Removed from Analysis)	N (after Removing Outliers)	ANOVA (Early Life Stress)	ANOVA (Sex)	ANOVA (Early Life Stress × Sex Interaction)
Substantia Nigra					
*Nr3c1* (GR)	-	46	F(1,42) = 3.78, *p* = 0.06	F(1,42) = 0.17, *p* = 0.68	F(1,42) = 0.03, *p* = 0.87
*Fkbp5*	Stress (1)	45	F(1,41) = 0.70, *p* = 0.41	**F(1,41) = 4.17, *p* = 0.05**	**F(1,41) = 6.33, *p* = 0.02**
*Fkbp4*	Control (1)	45	F(1,41) = 2.70, *p* = 0.11	**F(1,41) = 6.38, *p* = 0.02**	F(1,41) = 0.12, *p* = 0.73
*Ptges3*	Control (3)	43	F(1,39) = 1.41, *p* = 0.24	F(1,39) = 2.33, *p* = 0.14	F(1,39) = 0.40, *p* = 0.53
*Bag1*	-	46	F(1,42) = 2.18, *p* = 0.15	F(1,42) = 1.16, *p* = 0.29	F(1,42) = 1.19, *p* = 0.28
VTA					
*Nr3c1* (GR)	-	46	F(1,42) = 1.64, *p* = 0.21	F(1,42) = 0.19, *p* = 0.67	**F(1,42) = 7.87, *p*= 0.008**
*Fkbp5*	Stress (2)	44	**F(1,40) = 7.27, *p* = 0.01**	F(1,40) = 0.40, *p* = 0.53	F(1,40) = 0.16, *p* = 0.69
*Fkbp4*	Stress (1)	45	F(1,41) = 0.11, *p* = 0.75	F(1,41) = 0.03, *p* = 0.85	F(1,41) = 0.02, *p* = 0.90
*Ptges3*	-	46	**F(1,42) = 4.24, *p* = 0.05**	F(1,42) = 1.20, *p* = 0.28	F(1,42) = 1.13, *p* = 0.29
*Bag1*	-	46	**F(1,42) = 10.25, *p* = 0.003**	F(1,42) = 0.82, *p* = 0.37	**F(1,42) = 7.66, *p* = 0.008**
Dorsal Striatum					
*Nr3c1* (GR)	-	46	F(1,42) = 0.71, *p* = 0.40	F(1,42) = 0.007, *p* = 0.93	F(1,42) = 0.90, *p* = 0.35
*Fkbp5*	-	46	F(1,42) = 1.28, *p* = 0.27	F(1,42) = 0.36, *p* = 0.55	F(1,42) = 0.03, *p* = 0.86
*Fkbp4*	-	46	F(1,42) = 1.80, *p* = 0.19	**F(1,42) = 7.55, *p* = 0.009**	F(1,42) = 0.23, *p* = 0.63
*Ptges3*	Stress (1)	45	F(1,41) = 0.28, *p* = 0.60	F(1,41) = 0.001, *p* = 0.98	F(1,41) = 0.03, *p* = 0.86
*Bag1* *	-	46	F(1,42) = 0.23, *p* = 0.64	F(1,42) = 0.08, *p* = 0.78	F(1,42) = 2.07, *p* = 0.16
Ventral Striatum					
*Nr3c1* (GR)	-	46	F(1,42) = 0.009, *p*= 0.93	F(1,42) = 0.04, *p* = 0.85	F(1,42) = 2.78, *p* = 0.10
*Fkbp5*	-	46	F(1,42) = 1.39, *p* = 0.25	F(1,42) = 0.76, *p* = 0.39	F(1,42) = 0.14, *p* = 0.72
*Fkbp4*	Control (1)	45	F(1,41) = 0.001, *p* = 0.98	F(1,41) = 1.77, *p* = 0.19	F(1,41) = 1.78, *p* = 0.19
*Ptges3*	Stress (2)	44	F(1,40) = 1.23, *p* = 0.28	F(1,40) = 0.67, *p* = 0.42	F(1,40) = 0.11, *p* = 0.74
*Bag1*	-	46	F(1,42) = 2.60, *p* = 0.11	F(1,42) = 0.25, *p* = 0.62	F(1,42) = 0.32, *p* = 0.57

**Table 2:** Two-way ANOVA showing main effects of sex, treatment and interaction between sex and treatment on *Bdnf* transcript levels in the ventral striatum of 22 control and 24 maternally separated rodents. Asterisks (*) indicate genes whose log-transformed data were used for statistical analysis. Bold type indicates statistically significant effects.

Transcripts	Outliers (Removed from Analyses)	N (after Removing Outliers)	ANOVA (Early Life Stress)	ANOVA (Sex)	ANOVA (Early Life Stress × Sex Interaction)
Substantia Nigra					
*Bdnf I*	-	46	F(1,42) = 0.34, *p* = 0.56	**F(1,42) = 21.51, *p* < 0.001**	F(1,42) = 1.86, *p* = 0.18
*Bdnf IIA*	-	46	**F(1,42) = 10.93, *p* = 0.002**	F(1,42) = 1.88, *p* = 0.18	F(1,42) = 0.45, *p* = 0.50
*Bdnf IIC*	-	46	F(1,42) = 0.32, *p* = 0.58	**F(1,42) = 8.52, *p* = 0.006**	F(1,42) = 0.005, *p* = 0.94
*Bdnf III*	-	46	**F(1,42) = 22.54, *p* < 0.001**	F(1,42) = 0.41, *p* = 0.53	F(1,42) = 0.41, *p* = 0.52
*Bdnf IV*	-	46	F(1,42) = 0.51, *p* = 0.48	F(1,42) = 3.11, *p* = 0.09	F(1,42) = 2.28, *p* = 0.14
*Bdnf VI*	-	46	F(1,42) = 0.06, *p* = 0.82	**F(1,42) = 7.12, *p* = 0.01**	F(1,42) = 0.27, *p* = 0.60
(VTA					
*Bdnf I* *	-	46	F(1,42) = 2.02, *p* = 0.16	**F(1,42) = 10.46, *p* = 0.002**	F(1,42) = 1.64, *p* = 0.21
*Bdnf IIA*	-	46	**F(1,42) = 11.61, *p* = 0.001**	F(1,42) = 0.65, *p* = 0.43	F(1,42) = 0.91, *p* = 0.34
*Bdnf IIC*	-	46	F(1,42) = 1.51, *p* = 0.23	**F(1,42) = 4.08, *p* = 0.05**	F(1,42) = 1.05, *p* = 0.31
*Bdnf III*	-	46	**F(1,42) = 11.18, *p* = 0.002**	F(1,42) = 1.25, *p* = 0.27	F(1,42) = 0.17, *p* = 0.69
*Bdnf IV*	Stress (1)	45	F(1,41) = 1.59, *p* = 0.21	F(1,41) = 1.79, *p* = 0.19	F(1,41) = 2.58, *p* = 0.12
*Bdnf VI*	Stress (1)	45	F(1,41) = 0.89, *p* = 0.35	F(1,41) = 0.41, *p* = 0.53	F(1,41) = 3.17, *p* = 0.08
Dorsal Striatum					
*Bdnf I* *	Stress (1)	45	F(1,41) = 0.27, *p* = 0.61	F(1,41) = 0.19, *p* = 0.67	F(1,41) = 1.02, *p* = 0.32
*Bdnf IIA*	Stress (3), Control (1)	42	F(1,38) = 0.22, *p* = 0.65	F(1,38) = 0.09, *p* = 0.77	F(1,38) = 0.32, *p* = 0.57
*Bdnf IIC*	Stress (1), Control (1)	44	F(1,40) = 0.05, *p* = 0.83	F(1,40) = 1.97, *p* = 0.17	F(1,40) = 0.87, *p* = 0.36
*Bdnf III*	Stress (1)	45	F(1,41) = 0.45, *p* = 0.51	F(1,41) = 1.20, *p* = 0.28	F(1,41) = 1.61, *p* = 0.21
*Bdnf IV*	Stress (2)	44	F(1,40) = 0.02, *p* = 0.89	F(1,40) = 1.19, *p* = 0.28	F(1,40) = 0.98, *p* = 0.33
Bdnf VI*	Stress (1)	45	F(1,41) = 0.28, *p* = 0.60	F(1,41) = 2.58, *p* = 0.12	F(1,41) = 0.57, *p* = 0.45
Ventral Striatum					
*Bdnf I*	Stress (2)	44	**F(1,40) = 10.08, *p* = 0.003**	F(1,40) = 1.36, *p* = 0.25	F(1,40) = 1.22, *p* = 0.28
*Bdnf IIA* *	Stress (1)	45	**F(1,41) = 5.48, *p* = 0.02**	F(1,41) < 0.001, *p* = 0.98	F(1,41) = 0.14, *p* = 0.72
*Bdnf IIC*	Stress (1)	45	**F(1,41) = 6.48, *p* = 0.02**	F(1,41) = 0.18, *p* = 0.68	F(1,41) = 0.05, *p* = 0.83
*Bdnf III*	Stress (2)	44	F(1,40) = 3.11, *p* = 0.09	F(1,40) = 0.79, *p* = 0.38	F(1,40) = 0.07, *p* = 0.80
*Bdnf IV* *	-	46	**F(1,42) = 6.26, *p* = 0.02**	F(1,42) = 2.09, *p* = 0.16	F(1,42) = 0.24, *p* = 0.63
*Bdnf VI*	Stress (1)	45	**F(1,41) = 7.43, *p* = 0.009**	F(1,41) = 0.76, *p* = 0.39	F(1,41) = 0.84, *p* = 0.37

## Data Availability

The data presented in this study are available upon request from the corresponding author.
